# Rhinovirus Biology, Antigenic Diversity, and Advancements in the Design of a Human Rhinovirus Vaccine

**DOI:** 10.3389/fmicb.2017.02412

**Published:** 2017-12-05

**Authors:** Christopher C. Stobart, Jenna M. Nosek, Martin L. Moore

**Affiliations:** ^1^Department of Biological Sciences, Butler University, Indianapolis, IN, United States; ^2^Department of Pediatrics, Emory University, Atlanta, GA, United States; ^3^Children’s Healthcare of Atlanta, Atlanta, GA, United States

**Keywords:** rhinovirus, vaccine, common cold, respiratory disease, viral diversity

## Abstract

Human rhinovirus (HRV) remains a leading cause of several human diseases including the common cold. Despite considerable research over the last 60 years, development of an effective vaccine to HRV has been viewed by many as unfeasible due, in part, to the antigenic diversity of circulating HRVs in nature. Over 150 antigenically distinct types of HRV are currently known which span three species: HRV A, HRV B, and HRV C. Early attempts to develop a rhinovirus vaccine have shown that inactivated HRV is capable of serving as a strong immunogen and inducing neutralizing antibodies. Yet, limitations to virus preparation and recovery, continued identification of antigenic variants of HRV, and logistical challenges pertaining to preparing a polyvalent preparation of the magnitude required for true efficacy against circulating rhinoviruses continue to prove a daunting challenge. In this review, we describe HRV biology, antigenic diversity, and past and present advances in HRV vaccine design.

## Introduction

Human rhinoviruses (HRVs) are respiratory viruses that belong to the family *Picornaviridae* and genus *Enterovirus*. HRVs were first isolated in the 1950s and remain the leading cause of upper respiratory tract infections worldwide (Price, 1956; Jacobs et al., 2013). HRV remains the primary etiologic agent of the common cold and is responsible for significant morbidity, medical costs, and absences from school and the workplace (Fendrick et al., 2003; Pappas et al., 2008; Gern, 2010; van der Zalm et al., 2011; Jacobs et al., 2013). Annual economic costs associated with HRV infections are likely to exceed 60 billion dollars per year (Fendrick et al., 2003). HRV infections occur year-round and in all parts of the world and exhibit seasonality, peaking during the spring and/or autumn (Winther et al., 2006; Miller et al., 2007; Jacobs et al., 2013; Litwin and Bosley, 2014; Leotte et al., 2017). In addition to causing upper respiratory infections, HRV has also been linked to development and shown to exacerbate asthma and lower respiratory tract infections in human asthmatic volunteers (Hicks et al., 2006; Malmstrom et al., 2006; Renwick et al., 2007; Jackson et al., 2008, 2014; Message et al., 2008; Gern, 2010; Jacobs et al., 2013; Saraya et al., 2014). The significant health burden associated with HRV continues to highlight the dire need for an HRV vaccine. Yet, several challenges have precluded efficacious vaccine development.

## HRV Infections and Disease

Rhinoviruses are among the most common viral infectious agents found in humans. HRVs account for more then 50% of upper respiratory tract infections and infection rates among young children can be as high as 8–12 times a year (Arruda et al., 1995; Turner, 1997; Pappas et al., 2008). HRV infections are generally associated with an incubation period of 2 days followed by a symptomatic period of 1 to 2 weeks before clearance (Pappas et al., 2008). Asymptomatic infections of the nasopharynx are quite common for rhinoviruses, especially among young children (van Benten et al., 2003; Iwane et al., 2011). Upper respiratory tract infections are associated with common cold-like symptoms including rhinorrhea, sore throat, coughing, sneezing, nasal congestion, and general malaise (Arruda et al., 1997; Jacobs et al., 2013). In addition to the common cold, rhinovirus infections are often linked to acute otitis media and rhinosinusitis, which also frequently coincide with bacterial coinfection (Pitkaranta et al., 1997; Alper et al., 2007; Jackson et al., 2008; Jacobs et al., 2013). Despite an optimal temperature for HRV replication in the cooler surfaces (32–33°C) of the upper respiratory tract, rhinoviruses are also implicated with lower respiratory diseases including pneumonia, bronchitis, and bronchiolitis, and exacerbation of asthma (McFadden et al., 1985; Turner, 1997; Papadopoulos et al., 2000; Renwick et al., 2007; Gern, 2010). Over half of all asthma exacerbation incidents are known to be associated with HRV infections and early HRV infections resulting in wheezing promote a greater risk of asthma development later in life (Gern and Busse, 1999; Gern et al., 1999; Hayden, 2004; Jackson et al., 2008; Dougherty and Fahy, 2009). Approximately, 90% of children hospitalized with acute asthma attacks were shown to have detectable HRV (Bizzintino et al., 2011). Furthermore, a study evaluating 3 to 18-year-old patients admitted with wheezing identified nearly half tested positive for HRV (Heymann et al., 2004). Collectively, these studies demonstrate that HRV, though not typically considered a pathogen of high mortality, has a high potential for acute respiratory illness and the potential to promote or exacerbate chronic respiratory health conditions.

Transmission of HRVs occurs primarily between individuals by either direct contact, contact with fomites, or aerosols (Gwaltney and Hendley, 1978; Gwaltney et al., 1978; Dick et al., 1987; Jennings and Dick, 1987; Hendley and Gwaltney, 1988). Rhinoviruses are capable of remaining infectious on surfaces outside of hosts for hours resulting in a high potential for spread between infected and uninfected individuals (Hendley et al., 1973). Susceptibility to severe infection and re-infections has been linked to many environmental and genetic factors including poor interferon responses, existing allergies or asthma, exposure to air pollutants including tobacco smoke, poor diet, and stress (Gern, 2010; Kang et al., 2012; Jacobs et al., 2013). Rates of reinfection with specific types of HRV suggest that in some cases of HRV infection, competent immunity failed to develop or that pre-existing immunity cannot be maintained (Papi et al., 2006).

## HRV Structure and Biology

**FIGURE 1:**
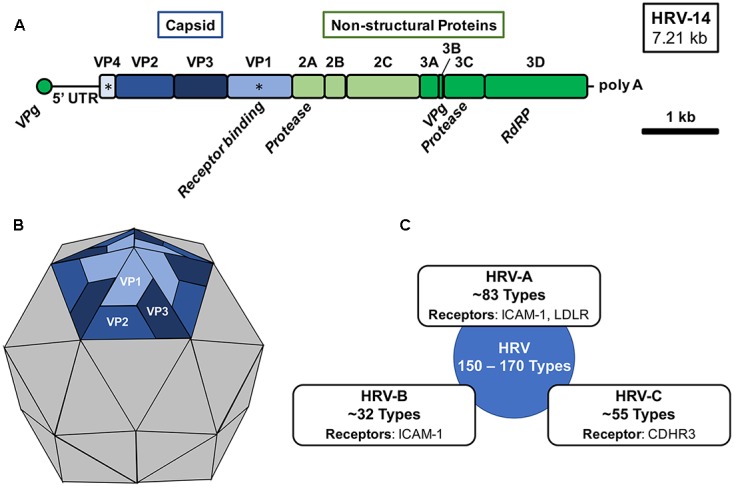
Human rhinovirus genomic organization, virion structure, and species. **(A)** The 7.21 kb +ssRNA genome of HRV-16 is comprised of a single open-reading frame encoding 11 gene products, which upon translation into three distinct polyproteins are cleaved by HRV-encoded proteases (2A and 3C). The 5′-end of the genome is capped with a short viral priming protein (VPg) for incorporation during virion assembly and a the 3′-end is polyadenylated. Capsid proteins VP1 and VP4 (^∗^) are generally used for phylogenetic analysis. RdRP, RNA-dependent RNA polymerase; UTR, untranslated region. **(B)** An icosahedral virion structure of HRV with a pentamer structure shown highlighting the external capsid proteins (VP1, VP2, and VP3) organization. VP1 is responsible for receptor engagement and VP4 is located beneath each monomeric unit and is responsible for genomic association with VPg. **(C)** Three distinct species of human rhinovirus have been identified (HRV-A, HRV-B, and HRV-C). The approximate number of types within each species classification and known receptors of each are shown.

Human rhinovirus is a non-enveloped virus with a positive-sense single-stranded RNA (+ssRNA) of approximately 7.2 kb that encodes 11 proteins (**Figure 1A**) (Palmenberg et al., 2009, 2010; Palmenberg and Gern, 2015). The viral capsid of HRV is comprised of four viral proteins (VPs): VP1, VP2, VP3, and VP4. The remaining viral proteins are responsible for viral replication and subsequent assembly. Antigenic variation among HRV types is derived from variations in the exposed surface of VP1, VP2, and VP3, while embedded VP4 is responsible for RNA packaging during assembly (**Figure 1B**). Compared to the rest of the HRV genome, the capsid proteins exhibit a high degree of heterogeneity resulting in a wide range of antigenic diversity (Glanville and Johnston, 2015; Lewis-Rogers et al., 2017). Several antigenic sites have been identified for HRV strains through study of binding of neutralizing antibodies. However, the locations of these sites are often not conserved (Sherry and Rueckert, 1985; Sherry et al., 1986; Appleyard et al., 1990).

VP1 mediates cell surface attachment through engagement of a variety of cell surface receptors (**Figure 1C**) (Palmenberg and Gern, 2015; Blaas, 2016). Traditionally, the majority of HRV types were known to bind the intercellular adhesion molecule 1 (ICAM-1) receptor and a minority of HRV types utilized the low-density lipoprotein receptor (LDLR) for binding (Staunton et al., 1989; Hofer et al., 1994; Palmenberg, 2017). However, with the recent identification of a specific variant of cadherin-related family member 3 (CDHR3) as the primary receptor for HRV C species and the breadth of circulating HRV types, yet-to-be identified receptors may also exist (Bochkov et al., 2015, 2016; Palmenberg, 2017). HRV may enter the cell through several pathways including macropinocytosis and clathrin-dependent and clathrin-independent endocytosis (Blaas, 2016). Upon entry and uncoating, the viral genome is translated and subsequently proteolytically processed by virus-encoded proteases, 2A and 3C (Fuchs and Blaas, 2010). During virion assembly and genomic packaging, 60 units of each capsid protein associate, with 1 unit of each capsid protein per face, to form the icosahedral structure encapsulating the RNA genome of the virus (Rossmann et al., 1985; Palmenberg et al., 2010; Liu et al., 2016).

## Phylogenetic Diversity of Rhinoviruses

Three species of rhinovirus are currently known: HRV-A, HRV-B, and HRV-C. However, sequencing and serologic methods have defined approximately 83 HRV-A types, 32 HRV-B types, and 55 HRV-C types with potentially as many as 150 to 170 serological distinct HRV types in circulation (**Figure 1C**) (Palmenberg et al., 2009; McIntyre et al., 2013). Rhinovirus strains within a given species share greater than 70% amino acid identity and new types are classified almost exclusively now based on VP1 or VP4/VP2 sequence alignments (Palmenberg and Gern, 2015). Isolates which share greater than 87% identity may be merged with existing types (McIntyre et al., 2013; Palmenberg and Gern, 2015). A major challenge to the development of an HRV vaccine and subsequent establishment of protective immunity is the phylogenetic breadth of existing HRV serotypes. Infection with one type of HRV is unlikely to afford any immunity to other types resulting in lifelong infections to different HRV type exposures. However, limited cross-serotype protection has been demonstrated for some closely-related types (Cooney et al., 1973; Glanville and Johnston, 2015). Among known types of HRV, HRV-A, and HRV-C are generally associated with more severe disease and asthma exacerbations than HRV-B (Miller et al., 2009; Piralla et al., 2009; Bochkov et al., 2011; Lee et al., 2012, 2016). Structures of all three species exhibit distinct surface pocket differences which may account for their phenotypic differences (Palmenberg and Gern, 2015; Liu et al., 2016).

## Recent Discovery and Biology of HRV-C

HRV-C was recently identified in 2006 and has since been recognized as a major contributor to HRV disease (Arden et al., 2006; Lamson et al., 2006; Kistler et al., 2007; Renwick et al., 2007; Dominguez et al., 2008). Over half of all HRV infections in young infants are now recognized as HRV-C strains (Bochkov et al., 2011). Furthermore, among HRV types detected during childhood acute asthma episodes, HRV-C was the more frequently detected species (Bizzintino et al., 2011). Following their initial identification, nearly 60 types have been identified and they have now been classified as a distinct species of HRV from HRV-A and HRV-B (Simmonds et al., 2010; McIntyre et al., 2013; Palmenberg and Gern, 2015). However, unlike HRV-A and HRV-B, study of HRV-C strains has been complicated by the inability to initially grow these viruses in cell culture.

The first report to describe successful recovery of an HRV-C type (C15) via reverse genetics was in 2011, however these viruses could not be subsequently passaged (Bochkov et al., 2011). Unlike its counterparts, HRV-C types do not use ICAM-1 or LDLR as a primary receptor for attachment (Bochkov et al., 2011). Phenotypically, HRV-C may be more tolerable of higher temperatures for replication resulting in more lower respiratory tract disease than species A or B (Ashraf et al., 2013). Furthermore, the virus could not be readily passaged or cultured until the recent identification of CDHR3 as a primary receptor of the virus in 2015 (Bochkov et al., 2015). The first capsid structure of an HRV-C type soon followed (Liu et al., 2016). Combined with a now cultivable reverse genetics approach, considerable attention is being given to study HRV-C biology. Recent work has shown that HRV-C selectively targets ciliated airway epithelial cells which express CDHR3 (Griggs et al., 2017). These findings are encouraging and pave the way for more thorough study of HRV-C biology and potential development of HRV-C-specific antivirals and vaccines. Due to its significant role in human disease, development of an effective rhinovirus vaccine for the common cold will need to include antigenic coverage from representative HRV-C strains.

## Challenges to Design of HRV Vaccines

Development of a vaccine for rhinovirus remains a universal hope of the public health and scientific communities. For over a half century, the prospect of developing an HRV vaccine has remained bleak. There remain many technical, logistical, and fundamental biological challenges to developing a successful vaccine for HRV. Mice and cottons rats are important models for testing of efficacy of vaccines to elicit neutralizing antibodies, however, they are not fully permissive to infection and can only resemble some aspects of HRV pathogenesis in humans (Bartlett et al., 2008; Blanco et al., 2014a). Recent advances in producing mice that are transgenic for human ICAM-1 have made them an improved model for study of HRV infection (Bartlett et al., 2008). Yet, any vaccine preparation would likely have to be studied directly in humans.

Unlike many other human viral pathogens, the shear breadth of serologically distinct types of HRV presents a unique and difficult challenge to vaccine design. In order to develop an HRV vaccine, regardless of approach (e.g., live-attenuated, inactivated), one or more vaccination preparations must be able to elicit protective neutralizing antibodies to potentially over 150 serologically distinct types spanning three different species. Despite advances in cell culture resulting in increased titers of virus, the shear technical challenge of producing sufficient quantity and quality of antigen to cover the breadth of HRV types remains a key technical challenge. In addition, there remains insufficient surveillance and epidemiologic data to accurately identify which specific dominant HRV-A and HRV-C types to prioritize in vaccine preparations to mitigate disease. Furthermore, several studies using both antivirals, antigenic peptide preparations, and virus have reported the induction of escape mutations, often with a single amino acid (Heinz et al., 1989; Appleyard et al., 1990; Speller et al., 1993). However, the structure of the majority of HRV types have yet to be resolved and only recently has the first HRV-C structure been determined (Liu et al., 2016). Despite these clear challenges, there have been advances in understanding how to develop an HRV vaccine and the feasibility of inducing lasting protective immunity.

## Past and Current Vaccine Efforts

By the 1960s, it became evident that many serologically distinct strains of rhinovirus existed and that development of a polyvalent vaccine preparation would be required to combat the common cold (Mitchison, 1965). Early attempts to develop an HRV vaccine during the 1960 and 1970s focused on: (1) whether live-attenuated or inactivated HRV strains induced antibodies and were capable of affording protection; (2) if heterologous or divergent strains could induce broad protection and lastly, (3) if multivalent vaccine preparations could be efficacious. A study by Bynoe et al. (1961) demonstrated that individuals were capable of having protective levels of antisera to a specific strain of HRV. Two follow-up studies published in 1963 demonstrated that a single strain of either live-unattenuated HRV, or HRV inactivated by formalin or heat, was capable of inducing antibodies following intramuscular injection and that circulating antibody levels correlated inversely with associated illness (Doggett et al., 1963; Mufson et al., 1963). By Mitchison (1965), it was evident that vaccination with a strain of HRV was capable of not only inducing protective antibodies, but also may provide homologous protection to the same strain. Subsequent studies published over the next few years supported that HRV vaccines could be efficacious with lasting immunity following homologous challenge, but not with heterologous strains (Perkins et al., 1969a,b; Buscho et al., 1972).

In the early 1970s, the first studies evaluating cross-neutralization and serologic reactivity between many HRV types were published which opened the door for the earliest attempts at polyvalent vaccine preparations. Cooney et al. (1973) demonstrated that rabbit antisera to 37 types resulted in limited cross-neutralization between at least five types. These studies and earlier studies using monovalent preparations led to the first major attempt at a polyvalent vaccine for HRV in Hamory et al. (1975). By this time, 89 HRV types had been described and several others were in varying stages of confirmation. In this study, Hamory et al. (1975) produced two separate decavalant HRV vaccine preparations by combining 10 formalin-inactivating unique sets of types with initial titers of 10^1.5^–10^6.0^ TCID_50_/mL. Unfortunately, these vaccine preparations resulted in low titers of neutralizing antibodies with detectable host responses to less than 40% of input types. Compared to the known number of types at the time, development of an effective polyvalent vaccine was soon regarded as improbable or impossible (Fox, 1976; Couch, 1984).

Over the next 30 years, scientific advances in cellular and molecular biology and continued research on HRV biology and type-specific differences led to renewed interest and hope in development of an HRV vaccine. The first capsid HRV crystal structure (HRV-14) was resolved in Rossmann et al. (1985). Structures of all three species of HRV would soon be determined with over 70 structures now deposited in the protein data bank (PDB) (Palmenberg, 2017). In Palmenberg et al. (2009, 2010), full genomic sequences were published for a significant number of known HRV-A and HRV-B strains, which provided new insight into HRV evolution, specific-strain differences, and strong evidence for recombination of antigenic types.

In light of the daunting task of developing a polyvalent inactivated vaccine preparation to over 100 types and advances in understanding HRV structure, recent efforts have focused on identifying and exploiting conserved antigenic sites through subunit and/or T-cell based approaches. Several recent attempts using VP1 as an immunogen have demonstrated that cross-serotype reactive antibodies can be generated with enough exposure and recombinant VP1 may be a potential antigen for induction of limited cross-reactivity (Edlmayr et al., 2011; McLean et al., 2012). Furthermore, studies have also shown that N-terminus of VP4 is capable of eliciting cross-serotype antibodies (Katpally et al., 2009; Panjwani et al., 2016). Efforts in the field of influenza have shown that promoting recognition and immunity based on internal epitopes expressed to T cells can be effective at targeting more conserved epitopes and afford greater Th1 activity and cross-serotype protection (Lee et al., 2008; Hillaire et al., 2011). Earlier studies with HRV have demonstrated that T cell populations recognize both shared and type specific epitopes (Hastings et al., 1993a,b; Gern et al., 1997). Expression of a recombinant VP0 in combination with a Th1-focused adjuvant (combination of incomplete Fruend’s and CpG) was effective at promoting strong Th1-specific cross-serotype antibody responses (Glanville et al., 2013). These collective studies provide demonstrate that development of a subunit vaccine may be an effective approach to addressing HRV antigenic diversity in a vaccine preparation. In addition, these studies illustrate that cross-reactive antigens (with the use of specific adjuvants) may be used to increase serological breadth of HRV coverage while limiting the number of types of antigens necessary.

Efforts examining whole virus preparations continue to be used to explore the potential for vaccine preparations. In Blanco et al. (2014b), intramuscular administration with live HRV-16 was shown to induce cross-serotype neutralizing antibody responses after intranasal challenge in cotton rats. Last year, a 50-valent inactivated vaccine preparation comprised of types representative of the diversity of known HRV-A strains was shown to induce neutralizing antibodies to all but one type tested following boost in rhesus macaques (Lee et al., 2016). This study by Lee et al. (2016) also demonstrated using a reconstituted 1975 decavalent vaccine preparation from the Hamory et al. (1975) study, that the pitfall to this early polyvalent vaccine effort was likely a combination of inadequate antigen levels and the potential need for an adjuvant. These recent findings illustrated that a high valency in polyvalent vaccine preparations, as would likely be necessary in an HRV vaccine, may be effective at promoting lasting immunity provided that sufficient antigen levels are present.

## Closing Thoughts: The Future Potential for an HRV Vaccine

For much of the last century, an HRV vaccine was often regarded as impossible. A multivalent vaccine preparation for any pathogen with the valency necessary to afford protection for circulating HRV has yet to be demonstrated in any commercial vaccine. Yet, advances in our understanding of HRV types, capsid structure, and recent advances in identifying conserved cross-reactive antigenic sites have shed new light on directions to explore in vaccine design. Furthermore, advances in HRV reverse genetics and the potential to create chimeric virus preparations collectively suggest that development of an HRV vaccine is not technically impossible, but will require novel ingenuity and continued study of existing similarities and differences among HRV types.

## Author Contributions

Contributed directly to the writing and drafting, editing, and final approval of the article before submission CS, JN, and MM.

## Conflict of Interest Statement

MM co-founded Meissa Vaccines, Inc., and serves as the chief scientific officer for the company. MM is an inventor in a patent application (PCT/US2016/037658) describing a rhinovirus vaccine described herein. The other authors declare that the research was conducted in the absence of any commercial or financial relationships that could be construed as a potential conflict of interest.
